# Texas Vital Statistics

**Published:** 1910-03

**Authors:** 


TEXAS VITAL STATISTICS.—In January, 1910, the total number of births reported to the Texas Health Department was 3969. Males, 2097; females, 1842 (sex not given, 30); whites, 3601; blacks, 368; still-born, 125. In January there were thirty-four sets of twins born in the State. Number of deaths during January was 1700. Majority of births over deaths, 2269—over 100 per cent. Deaths by ages:

Under 1 year, 213; from 1 to 5, 154; from 5 to 10, 39; from 10 to 20, 89; from 20 to 30, 167; from 30 to 40, 197; from 40 to 50, 153; from 50 to 60, 186; over 60, 417; unknown, 85.

The causes of death show the following:

Pneumonia, 270; tuberculosis, 204; senile debility, 119; disease of infancy, 67; heart disease, 60; Bright's disease, 57; diarrhea and enteritis, 57; congestion and hemorrhage of brain, 38; typhoid, 37; stomach diseases, 37; broncho-pneumonia, 33; diphtheria and croup, 30; influenza, 30; paralysis, 27; acute nephritis, 25; peritonitis, 20; pellagra, 2.

No race suicide here.

